# Overexpression of *OsCIPK30* Enhances Plant Tolerance to *Rice stripe virus*

**DOI:** 10.3389/fmicb.2017.02322

**Published:** 2017-11-24

**Authors:** Zhiyang Liu, Xuejuan Li, Feng Sun, Tong Zhou, Yijun Zhou

**Affiliations:** ^1^Institute of Plant Protection, Jiangsu Academy of Agricultural Sciences, Jiangsu Technical Service Center of Diagnosis and Detection for Plant Virus Diseases, Nanjing, China; ^2^Scientific Observation and Experimental Station of Crop Pests in Nanjing, Ministry of Agriculture, Nanjing, China

**Keywords:** *Rice stripe virus*, NS3, interaction, OsCIPK30, pathogenesis-related genes

## Abstract

*Rice stripe virus* (RSV) causes a severe disease in *Oryza sativa* (rice) in many Eastern Asian countries. The NS3 protein of RSV is a viral suppressor of RNA silencing, but plant host factors interacting with NS3 have not been reported yet. Here, we present evidence that expression of RSV NS3 in *Arabidopsis thaliana* causes developmental abnormalities. Through yeast two-hybrid screening and a luciferase complementation imaging assay, we demonstrate that RSV NS3 interacted with OsCIPK30, a CBL (calcineurin B-like proteins)-interaction protein kinase protein. Furthermore, OsCIPK30 was overexpressed to investigate the function of OsCIPK30 in rice. Our investigation showed that overexpression of OsCIPK30 in rice could delay the RSV symptoms and show milder RSV symptoms. In addition, the expression of pathogenesis-related genes was increased in OsCIPK30 transgenic rice. These results suggest that overexpression of OsCIPK30 positively regulates pathogenesis-related genes to enhance the tolerance to RSV in rice. Our findings provide new insight into the molecular mechanism underlying resistance to RSV disease.

## Introduction

Rice stripe disease, caused by *Rice stripe virus* (RSV), is one of the most destructive diseases in rice (Wei et al., 2009). RSV is transmitted transovarially in a circulative manner by vector insects, primarily the small brown planthopper (SBPH; *Laodelphax striatellus*), exhibiting chlorotic and necrotic stripes in rice plant leaves followed by plant wilting (Hibino, 1996; Falk and Tsai, 1998). RSV was first reported in Japan in 1897 (Kisimoto, 1965) and is currently present in many East Asian countries, including China, where it was first reported in 1963. It is now widely spread in at least 16 different provinces of China (Wei et al., 2009).

*Rice stripe virus* belongs to the *Tenuivirus* genus, and the viral genome consists of four single-stranded RNA segments (Hibino, 1996; Falk and Tsai, 1998), which encode seven ORFs using a negative or ambisense coding strategy (Ramirez and Haenni, 1994). RNA1 is the largest RNA segment and has a single ORF in the viral-complementary sense, encoding an RNA-dependent RNA polymerase (RdRp) (Barbier et al., 1992; Toriyama et al., 1994). Each of the other three segments (RNA2, 3, and 4) has two have two open reading frames (ORFs), with one on the viral RNA (vRNA) and the other on the viral complementary strand (vcRNA) (Zhu et al., 1991, 1992; Takahashi et al., 1993). RSV vRNA2 encodes a membrane-associated protein (also named NS2) that has been reported to be an RNA silencing suppressor (Du et al., 2011). The vcRNA2 encodes a glycoprotein (also named NSvc2), which moves from the endoplasmic reticulum to the Golgi bodies via the coat protein complexes I – and coat protein complexes II-dependent secretion pathway (Zhao et al., 2012; Yao et al., 2014). vRNA3 encodes an RNA silencing suppressor (also named NS3), suppressing both local and systemic RNA silencing in the plant and vcRNA3 encodes a nucleocapsid protein (also named NP) from the vcRNA (Xiong et al., 2009; Shen et al., 2010). RSV vRNA4 encodes a disease-specific protein (also named SP) that accumulates in both infected plant and insect cells (Toriyama, 1986). The protein encoded by vcRNA4 (also named MP) has been identified as a cell-to-cell movement protein (Xiong et al., 2008).

Thus far, few host proteins have been reported to interact with RSV proteins. For example, DnaJ protein and a small heat-shock protein (HSP) of rice can interact with RSV MP, OsSGS3 (suppressor of gene silencing 3) can interact with NS2, and PsbP, a 23-kDa oxygen-evolving complex protein of plants, has been reported to interact with SP, resulting in the enhancement of virus symptoms (Lu et al., 2009; Du et al., 2011; Kong et al., 2014). Recently, host HSP70 and HSP20 have been shown to interact with RSV RdRp, the former of which is necessary for RSV infection and the latter of which can be altered by RSV for its expression pattern (Jiang et al., 2014; Li et al., 2015). Although NS3 was reported as a suppressor of RNA silencing, the host factors that may interact with NS3 are still unknown.

Plant CIPKs can be activated by interacting with calcineurin B-like proteins (CBLs) to transduce calcium signals by phosphorylating downstream signaling components, containing a Ser/Thr protein kinase domain in their N terminal region and an NAF domain [a novel protein interaction domain (first called by Albrecht et al., 2001)] in their C-terminal region (Luan, 2009). A systematic genome analysis revealed the existence of over 30 *OsCIPK* genes in rice (Kolukisaoglu et al., 2004; Xiang et al., 2007). Thus far, several studies have postulated the possible functions of OsCIPKs. OsCIPK19 is involved in the responses to light, nutrients, and low temperature (Ohba et al., 2000). OsCIPK23 is suggested to function in pollination and drought stress responses (Yang et al., 2008). The expression of OsCIPK31 is involved in diverse signals, such as cold, salt, light, cytokinins, and sugars (Kim et al., 2003). The mutant of OsCIPK31 exhibited hypersensitive phenotypes to abscisic acid, salt, mannitol, and glucose (Piao et al., 2010). Overexpression of OsCIPK3, OsCIPK12, and OsCIPK15 enhances tolerance to cold, drought, and salt stress, respectively (Xiang et al., 2007). OsCIPK15 has also been shown to play an important role in O_2_-deficiency tolerance in rice (Lee et al., 2009). To date, many *OsCIPKs* genes have been reported to be involved in response to abiotic stresses, but only few have been reported to be involved in responses to biotic stresses.

Here, we provide evidence showing that host OsCIPK30 interacts with RSV NS3, and that the overexpression of *OsCIPK30* in rice (*Oryza sativa* L. *japonica.* cv. Nipponbare) positively regulates pathogenesis-related genes to enhance tolerance to RSV.

## Materials and Methods

### Sources of Virus, Vectors, and Plant Materials

Rice plants infected with RSV were collected from Jiangsu province in China. Young instar nymphs of SBPHs were fed on the RSV-infected rice plants for 2 days to acquire the virus and were maintained on “Wuyujing No. 3” rice plants grown in an insect-rearing room at a temperature of 25 ± 3°C, 55 ± 5% relative humidity and under a light intensity of 200 μmol m^-2^ s^-1^ (16 h photoperiod). The proportions of viruliferous SBPHs were confirmed by dot-ELISA (Sun et al., 2011).

*Arabidopsis thaliana* (ecotype Columbia-0, Col-0) seeds were grown in potting soil in a growth chamber at 24°C under 200 μmol m^-2^ s^-1^ illumination. Rice plants used in this study were grown inside a growth chamber at 26°C, 80% relative humidity. Additionally, all plants were kept under 16-hlight/8-h dark photoperiod conditions.

### Inoculation of RSV

Rice plants (*Oryza sativa* L. spp. *japonica*. cv. Nipponbare) were inoculated with three viruliferous SBPHs per plant and were kept in a growth chamber. After inoculation for 3 days, planthoppers were removed back to rice field. And the inoculation does not hurt SBPHs at all, we just let them feed on the tested rice plants. Plants were maintained in a growth chamber for symptom development, and RSV-free SBPHs were used for mock inoculation.

### Generation of Transgenic Plants

Total RNA was extracted from RSV infected rice. cDNA synthesis and PCR were conducted as described previously (Sun et al., 2016) to get the full length of NS3. The full-length *NS3* DNA was integrated into the pENTR/D-TOPO vector (Invitrogen, Carlsbad, CA, United States) and was then cloned into the pBA-Flag-6Myc-DC binary vector (Zhang et al., 2005) using the LR clonase reaction mixture to induce constitutive expression of the *NS3* gene in *Arabidopsis*. The resultant 35S:*NS3* construct was introduced into *Agrobacterium tumefaciens* strain GV3101. Agrobacteria-mediated transformation of *A. thaliana* ecotype Col-0 with the *NS3* gene was conducted using the floral dip method (Clough and Bent, 1998; Zhang et al., 2006). The transgenic seeds were selected on standard MS medium (Murashige and Skoog, 1962) containing the appropriate antibiotics: 100 mg/L carbenicillin (Sangon Biotech, Shanghai, China).

Total RNA was extracted from *Oryza sativa* L. spp. *japonica*. cv. Nipponbare and the full-length *OsCIPK30* cDNA was cloned into the pUN1301 vector (Biovector, Co., Ltd, Beijing, China) to generate the Ubi-*OsCIPK30* plasmid. The construct was introduced into *A. tumefaciens* strain GV3101. Agrobacteria-mediated transformation protocols for rice were published previously (Wu et al., 2007). Regenerated transgenic plants (T_0_) were transplanted into soil; and the T_1_ progeny germinated from seeds were grown in soil inside a greenhouse. The T_1_ generations were confirmed by RT-PCR for testing *hygromycin* gene. Empty pUN1301 vector-transformed plants were used as the wild type (WT) controls.

### Alignment and Phylogenetic Analysis

Sequence alignment of 33 members of the OsCIPK family in rice was performed using ClustalW. Phylogenetic analysis of the 33 OsCIPKs in rice based on amino acid sequences carried out using a neighbor-joining (NJ) method with MEGA version 4.0.

### Yeast Two-Hybrid Assay

To construct the bait vector for the assay, the coding sequence of *NS3* was cloned into the yeast GAL4 binding domain vector pGBKT7, as instructed (Clontech, Mountain View, CA, United States). Construction and screening of a rice cDNA library (*Oryza sativa* L. spp. *japonica*. cv. Nipponbare) were performed as described in the BD Matchmaker Library Construction and Screening Kits User Manual (Clontech, Mountain View, CA, United States). Briefly, the rice cDNA library was screened using pGBKT7-NS3 as the bait in the *Saccharomyces cerevisiae* strain Gold (Clontech, Mountain View, CA, United States). The positive clones were selected on a histidine-deficient medium and then confirmed by β-gal assays. The coding sequence of *OsCIPK5* (Os01g0206700), *OsCIPK25* (Os06g0543400), *OsCIPK26* (Os02g0161000), *OsCIPK29* (Os07g0678300), *OsCIPK30* (Os01g0759200), and *OsCIPK32* (Os12g0132200) was PCR-amplified from rice leaf cDNA and then inserted into the yeast GAL4 activation domain vector, pGADT7 (Clontech, Mountain View, CA, United States).

Combinations of the plasmids BD-NS3 and AD-OsCIPK5, BD-NS3 and AD-OsCIPK25, BD-NS3 and AD-OsCIPK26, BD-NS3 and AD-OsCIPK29, BD-NS3 and AD-OsCIPK30, or BD-NS3 and AD-OsCIPK32 were co-transformed into the *S. cerevisiae* strain Gold. BD-53 and AD-T were also co-transformed into *S. cerevisiae* strain Gold to serve as a positive control. *S. cerevisiae* co-transformed with BD-Lam and AD-T, BD-NS3 and AD, or BD and AD-OsCIPK30 were as used as negative controls. All transformants were grown at 30°C for 72 h on synthetic medium lacking Leu and Trp and then transferred to medium lacking adenine, His, Leu, and Trp and supplemented with 0.5 mg ml^-1^ Aureobasidin A (AbA).

### Luciferase Complementation Imaging (LCI) Assay

The LCI was performed on 4-week-old *Nicotiana benthamiana* (*N. benthamiana*) leaves infiltrated with various combinations of *A. tumefaciens* GV3101 harboring pCambia-Myc-NS3-nLUC and pCambia-cLUC-3HA-candidate proteins. The agrobacteria containing the plasmids were incubated with 25 μM beta-estradiol for 3 h prior to infiltration, and all cultures were adjusted to OD_600_ = 0.8. The transfected leaves were assayed 2 days after agroinfiltration by adding the substrate (10 mM luciferin). The sprayed leaves were incubated in total darkness for 5 min and photographed using the Tanon 5200 Luminescent Imaging Workstation with an EMCCD camera. The images were processed with TanonImage software (Tanon Science & Technology, Shanghai, China).

### Western Blot Assay

Extraction of total protein from leaf tissues (100 mg per sample) for western blotting was done as described previously (Sun et al., 2016). The membranes were probed with anti-Myc antibodies (Sigma-Aldrich, St. Louis, MO, United States). The secondary antibody used in the study was a goat anti-rabbit IgG conjugated with alkaline phosphatase (Sigma-Aldrich, St. Louis, MO, United States).

### Real-Time Quantitative RT-PCR (qRT-PCR)

Equal amounts of the RNA samples from five independent plants of the same treatment were pooled. Total RNA was extracted from each treatment using RNAiso Plus reagent (Takara, Dalian, China), and cDNA synthesis and PCR were conducted as described previously (Sun et al., 2016). *Actin1* (Os03g0718100) was used as an internal standard. qRT-PCR was performed using the Bio-Rad iQ5 Real-time PCR System and SYBR Premix Ex Taq and the primers used in this assay are listed in **Supplementary Data Sheet 1** (Takara, Dalian, China). The comparative cycle threshold method was used to calculate the relative mRNA levels (Applied Biosystems, 1997; Johnson et al., 2000). All qRT-PCR experiments were performed at least three times.

### Statistical Analysis

Data were obtained from three replicates and analyzed by unpaired two-tailed Student’s *t*-test with GraphPad Prism software (GraphPad Software, Inc., La Jolla, CA, United States). Values of *P* < 0.05 or *P* < 0.01 were considered to indicate different levels of statistical significance.

## Results

### RSV NS3 Causes Developmental Abnormalities in *Arabidopsis*

**FIGURE 1:**
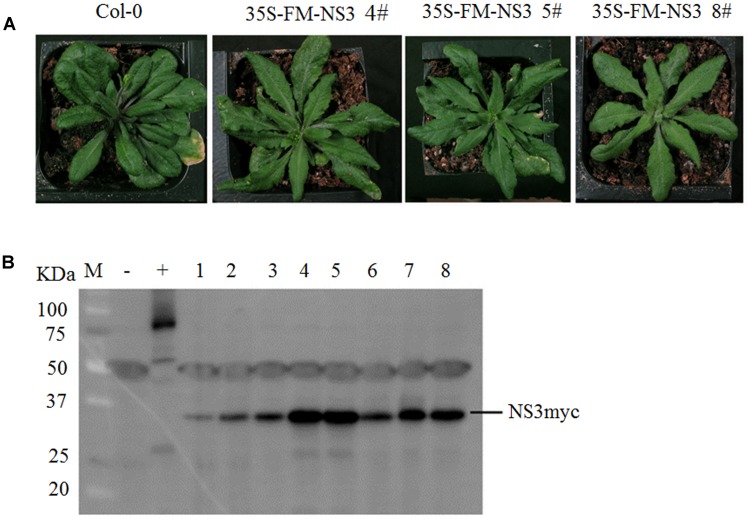
NS3 causes developmental abnormalities in *Arabidopsis.* **(A)** Morphological defects of *Arabidopsis* transgenic plants expressing RSV NS3. Photographs were taken 30 days after sowing. **(B)** Western blot analysis of 35S-Flag-6Myc-NS3 transgenic lines. The hyphen indicates the locations of the tagged NS3 proteins (23.87 KDa NS3 plus 7.2 KDa 6Myc). +: 35S-Flag-6Myc-SERRATE; –: *Col-0*; 1–8: individual NS3 transgenic lines.

To study the function of NS3, we generated transgenic *Arabidopsis* lines overexpressing full-length RSV NS3 with Flag-6Myc epitopes. These transgenic plants were confirmed by western blot assays (**Figure 1B**). Importantly, the majority of the transgenic lines exhibited developmental abnormalities with serrate true leaves (**Figure 1A**). The phenotypes of transgenic plants indicated that NS3 might impact growth and development in *Arabidopsis* and might also influence rice.

### RSV NS3 Interacts with OsCIPK30

**FIGURE 2:**
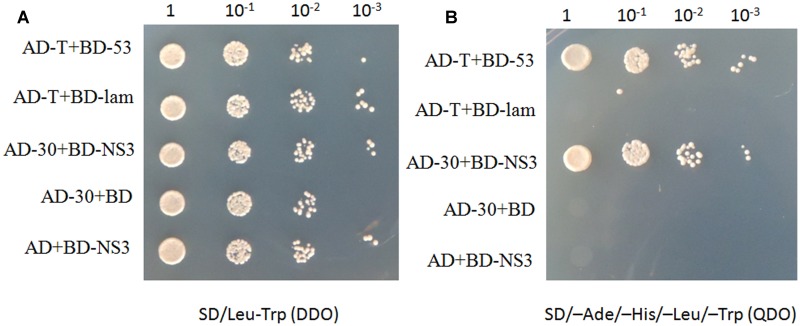
NS3 and OsCIPK30 interactions are detected in the yeast two-hybrid assay. Yeast strain Gold was co-transformed with the indicated plasmids shown on the left side of the panels. **(A)** The co-transformants were spread onto the SD/Leu-Trp (DDO) selection medium and **(B)** the SD/-Ade/-His/-Leu/-Trp (QDO) selection medium. AD-T+BD-53 were used as positive control, AD-T+BD-lam were used as negative control.

To identify plant host factor(s) that interact with NS3 during RSV infection, we screened a rice cDNA library using RSV NS3 as the bait through a yeast two-hybrid assay. Twenty-one positive cDNA clones were identified. One of them was a fragment of the OsCIPK30 gene. To test the interaction between the full-length OsCIPK30 and NS3, the ORFs of OsCIPK30 and NS3 were amplified and integrated into pGADT7 and pGBKT7, respectively. The recombinant plasmid (AD-30) was co-transformed into yeast cells with BD-NS3. **Figures 2A,B** show the positive interaction between the full-length OsCIPK30 and NS3 through yeast two-hybrid assays.

**FIGURE 3:**
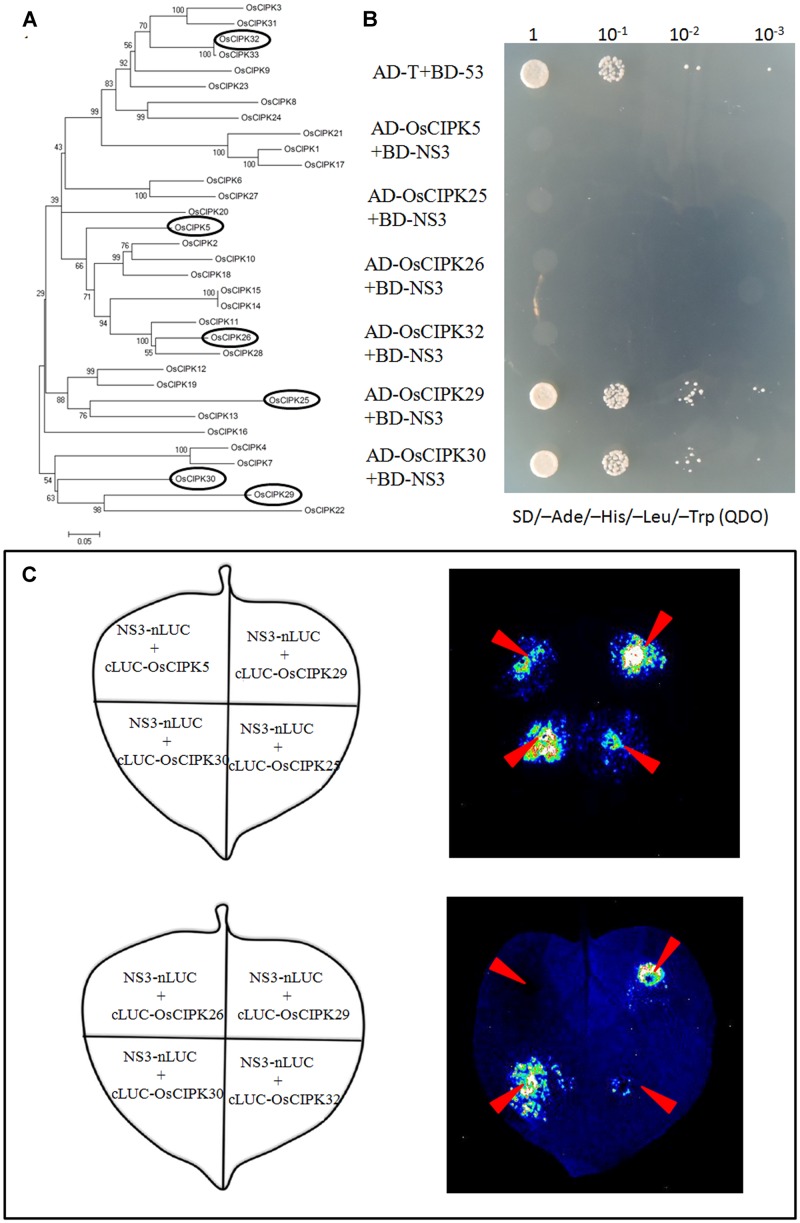
Phylogenetic analysis of OsCIPK proteins and the interaction between NS3 and OsCIPK5, OsCIPK25, OsCIPK26, OsCIPK29, OsCIPK30, and OsCIPK32 in yeast cells and plant cells. **(A)** Phylogenetic analysis of all 33 OsCIPK proteins. The analysis was performed using MEGA 4 software. The ellipses indicate the proteins chosen for the interaction assay. **(B)** The interaction between NS3 and OsCIPK5, OsCIPK25, OsCIPK26, OsCIPK29, OsCIPK30, and OsCIPK32 by yeast two-hybrid assay. The co-transformants were spread onto the SD/-Ade/-His/-Leu/-Trp (QDO) selection medium. **(C)** The interaction between NS3 and OsCIPK5, OsCIPK25, OsCIPK26, OsCIPK29, OsCIPK30, and OsCIPK32 by a luciferase complementation imaging (LCI) assay. Schematic representation of the LCI assay shows the different combinations of infiltrated constructs that were fused either to the N-terminal (NLuc) or C-terminal (CLuc) regions of luciferase. The infiltration positions of the constructs (red arrows) and luminescence signals resulting from the protein–protein interaction in a leaf are shown.

To demonstrate whether NS3 and OsCIPK30 also interacted in plant cells, a luciferase complementation imaging (LCI) assay was used. In the LCI experiments, the N- and C-terminal regions of firefly luciferase (NLuc and CLuc) were fused to NS3 and OsCIPK30, respectively, and were transiently expressed in *N. benthamiana*. When NLuc and CLuc are close to each other through the interaction of the two proteins, catalytic activity is restored and recorded through the CCD camera. In our LCI screening, NS3 displayed LUC complementation with OsCIPK30, suggesting that NS3 is physically close to OsCIPK30 *in vivo* (**Figure 3C**). Together, these assays clearly indicated that NS3 and OsCIPK30 interact *in vivo*.

As mentioned above, OsCIPK30 belongs to the CIPK family in rice. According to the Institute for Genomic Research [TIGR] database and NCBI database, we collected all 33 CIPK proteins in rice named from OsCIPK1 to OsCIPK33. Phylogenetic analysis of OsCIPKs showed that the proteins could be classified into several groups (**Figure 3A**); we chose the other five OsCIPK proteins from different groups and performed Y2H and LCI assays to identify whether they also interacted with NS3. The results showed that only OsCIPK29 was able to interact with NS3, whereas OsCIPK5, 25, 26, and 32 could not (**Figures 3B,C**).

### *OsCIPK30* Is Induced by RSV Infection

**FIGURE 4:**
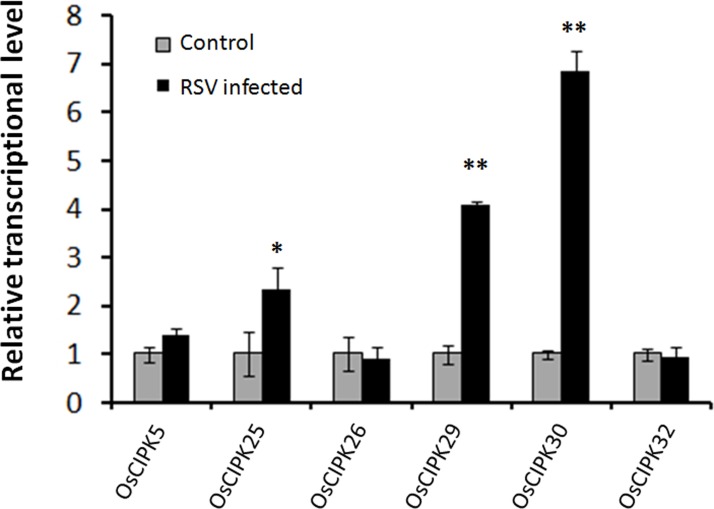
Expression of *OsCIPK* genes infected by RSV. The assay was performed by real-time RT-PCR. RNA was isolated from RSV-infected rice plants at 7 days post-inoculation. The relative mRNA levels were calculated with respect to the expression level of the corresponding transcript in the wild type (WT) rice plants without RSV infection. All the experiments were repeated three times, and similar results were obtained. The data represent the means ± SD of triplicate measurements. Asterisks above the columns represent significance based on an unpaired, two-tailed Student’s *t*-test relative to the WT. ^∗∗^*P* < 0.01; ^∗^*P* < 0.05.

To investigate whether RSV can induce the expression of *OsCIPKs* (*OsCIPK5, OsCIPK25, OsCIPK26, OsCIPK29, OsCIPK30*, and *OsCIPK32*), we examined the relative transcript level of these six *OsCIPK* genes in RSV-infected rice at 7 days post-inoculation (dpi). Real-time PCR showed that only three *OsCIPKs* were up-regulated, and two of them exhibited highly significant differences compared to the mock rice, suggesting that OsCIPK29 and OsCIPK30 might be involved in the host response to RSV infection (**Figure 4**).

### Overexpression of *OsCIPK30* Enhances the Tolerance to RSV

**FIGURE 5:**
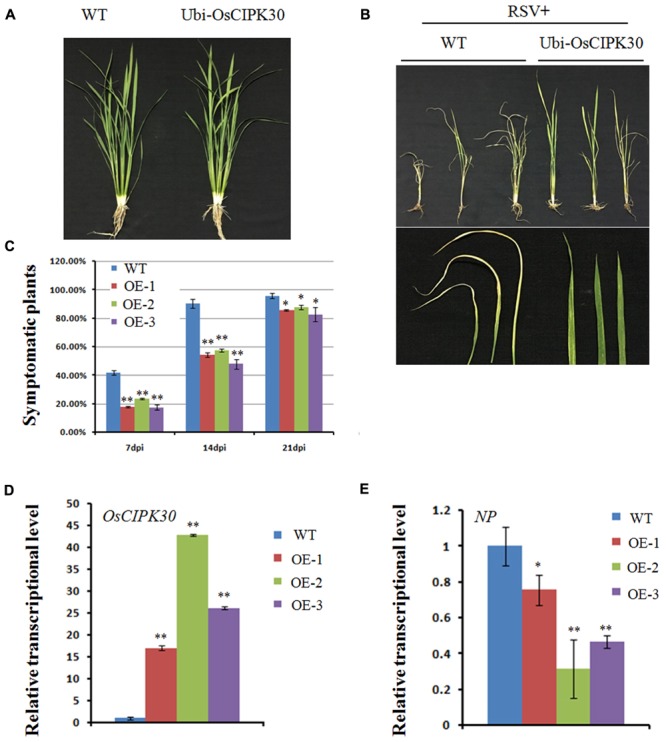
Effect of OsCIPK30 overexpression on RSV infection in rice. **(A)** Growth of the WT and Ubi-OsCIPK30 transgenic rice (T_1_ generation). **(B)** WT and Ubi-OsCIPK30 T_1_ transgenic rice were inoculated with RSV and photographed at 21 dpi. The lower panel of **(B)** shows the symptomatic leaves of the representative plants. **(C)** Temporal changes of RSV symptom development in WT and Ubi-OsCIPK30 T_1_ transgenic rice (OE-1, OE-2, OE-3). Thirty plants were used for each treatment in the experiment. **(D)** The expression level of *OsCIPK30* in T_1_ transgenic rice (OE-1, OE-2, OE-3) by real-time RT-PCR at 21 dpi with RSV. The relative mRNA levels were calculated with respect to the expression level of the corresponding transcript in the WT rice plant. **(E)** The expression level of *NP* in T_1_ transgenic rice (OE-1, OE-2, OE-3) by real-time RT-PCR at 21 dpi with RSV. The relative mRNA levels were calculated with respect to the expression level of the corresponding transcript in the WT rice plant. All the experiments were repeated three times, and similar results were obtained. The data represent the means ± SD of triplicate measurements. The asterisks above the columns represent significance based on an unpaired, two-tailed Student’s *t*-test relative to the WT. ^∗∗^*P* < 0.01; ^∗^*P* < 0.05.

Given that the transcription level of *OsCIPK30* is the highest in six *OsCIPKs* when RSV infects the host, we generated the overexpression transgenic rice of *OsCIPK30* to further investigate its function. Under greenhouse conditions, the Ubi-OsCIPK30 rice plants showed normal growth compared with WT (**Figure 5A**). We then inoculated WT and Ubi-OsCIPK30 transgenic rice plants with RSV and observed the symptom development of the plants over a time course.

Fewer transgenic rice plants showed symptoms than WT rice at 7 and 14 dpi; moreover, the Ubi-OsCIPK30 rice showed milder RSV symptoms than those in the WT rice at 21 dpi (**Figures 5B,C**). These results suggested that Ubi-OsCIPK30 rice could delay the RSV symptoms and might influence the RSV infection. The real-time PCR results demonstrated that the expression levels of *OsCIPK30* in transgenic rice plants were significantly increased, while the transcriptional level of the RSV *NP* gene was decreased (**Figures 5D,E**), indicating that the enhanced tolerance was associated with increased accumulation of *OsCIPK30* transcripts.

### Pathogenesis-Related Genes Are Up-regulated in *OsCIPK30-*Overexpressing Plants after RSV Infection

**FIGURE 6:**
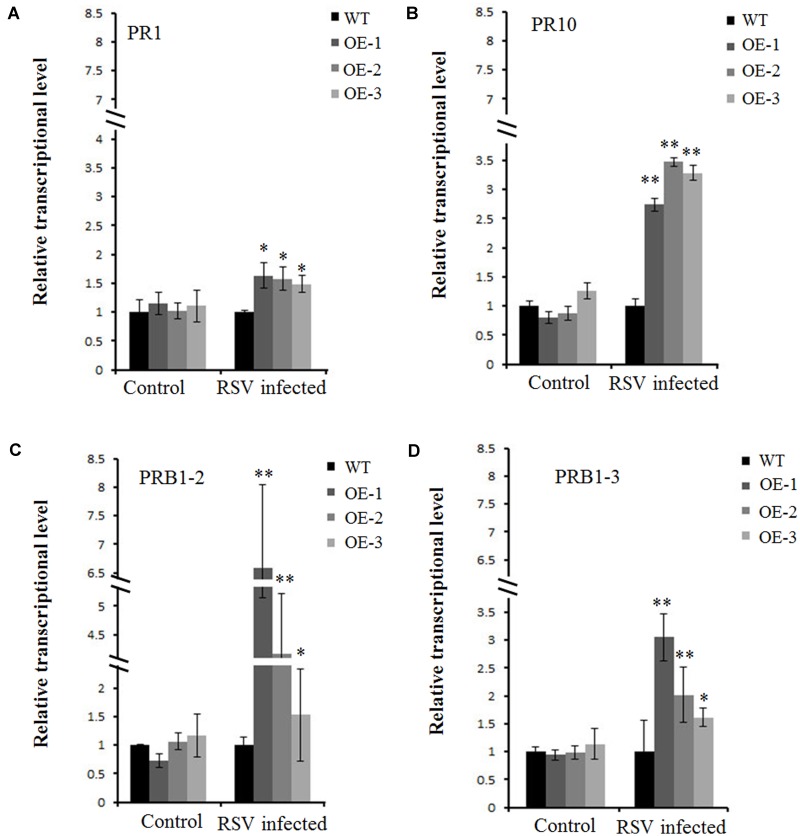
Transcriptionally modulated *OsCIPK30* influenced the expression of a set of pathogenesis-related genes. **(A)** The expression levels of PR10 were significantly higher in *OsCIPK30-*overexpressing rice plants (OE-1, OE-2, OE-3) than WT plants after RSV infection. **(B)** The expression levels of *PR1* were higher in *OsCIPK30-*overexpressing rice plants (OE-1, OE-2, OE-3) than WT plants after RSV infection. **(C)** The expression levels of PRB1-2 were significantly higher in *OsCIPK30-*overexpressing rice plants (OE-1, OE-2, OE-3) than WT plants after RSV infection. **(D)** The expression levels of PRB1-3 were significantly higher in *OsCIPK30-*overexpressing rice plants (OE-1, OE-2, OE-3) than WT plants after RSV infection. The un-inoculated plants were used as control. All experiments were repeated three times, and similar results were obtained. The data represent the means ± SD of triplicate measurements. Asterisks above the columns represent significance based on an unpaired, two-tailed Student’s *t*-test relative to the WT plants. ^∗∗^*P* < 0.01; ^∗^*P* < 0.05.

**FIGURE 7:**
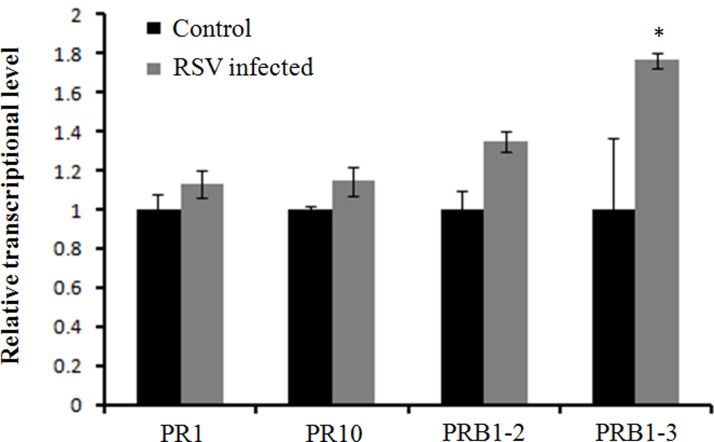
Expression of pathogenesis-related genes infected by RSV. The assay was performed by real-time RT-PCR. RNA was isolated from RSV-infected rice plants at 7 days post-inoculation. The relative mRNA levels were calculated with respect to the expression level of the corresponding transcript in the WT rice plants without RSV infection. All the experiments were repeated three times, and similar results were obtained. The data represent the means ± SD of triplicate measurements. Asterisks above the columns represent significance based on an unpaired, two-tailed Student’s *t*-test relative to the WT. ^∗∗^*P* < 0.01; ^∗^*P* < 0.05.

A previous study showed several pathogenesis-related genes involved in RSV infection (Satoh et al., 2010), and our results also showed that OsCIPK30 might influence RSV infection. To determine whether *OsCIPK30* is involved in pathogenesis-related defense systems, we analyzed the expression patterns of several pathogenesis-related genes. The second youngest leaves were collected from five independent plants of Ubi-OsCIPK30 rice and WT rice at 7 dpi. The real-time PCR showed that RSV infection strongly induced the expression of PR1, PR10, PRB1-2, and PRB1-3 in *OsCIPK30-*overexpressing plants, showing significant differences between transgenic plants and WT plants, while the expression levels of the PR genes were not changed in un-inoculated *OsCIPK30*-overexpressing plants compared with WT rice (**Figures 6A–D**). We also took an experiment showing the effect of the PR1, PR10, PRB1-2, and PRB1-3 expression in WT plants by RSV infection (**Figure 7**). The results showed that the expression levels of PRB1-3 increased after RSV infection in WT rice, but PR1, PR10, PRB1-2 not. Together with the results showing in **Figure 6**, we conclude that the pathogenesis-related genes are triggered in *OsCIPK30*-overexpressing plants after RSV infection. Taken together, these results suggest that *OsCIPK30* positively regulates the pathogenesis-related genes to enhance tolerance to RSV.

## Discussion

We detected and confirmed an interaction between RSV NS3 and OsCIPK29 and OsCIPK30 (**Figures 2**, **3**). To the best of our knowledge, this is the first report of plant host factors interacting with RSV NS3. NS3 is a viral suppressor of RNA silencing, and transgenic rice expressing NS3 could accumulate more RSV than in WT plants at the early stage of infection (Wu et al., 2014). In this study, we provided the transgenic *Arabidopsis* expressing NS3, which caused developmental abnormalities in plants (**Figure 1A**). All the results indicate that NS3 may be a pathogenic factor. Our results showed that NS3 could interact with OsCIPK29 and OsCIPK30 but failed to interact with other OsCIPK proteins. Given that OsCIPK genes have similar functional domains (the complete protein kinase domain and the NAF domain) (Xiang et al., 2007), the reason for the specific interaction between NS3 and OsCIPK29, 30 remains unclear.

As mentioned above, OsCIPK genes play some important roles in the response to abiotic stresses, such as drought, cold, salt, light, and sugars (Yu et al., 2014). In this study, OsCIPK30-overexpressing plants caused mild symptoms and induced high expression of pathogenesis-related genes (PR1, PR10, PRB1-2, and PRB1-3) upon RSV infection (**Figures 5**, **6**), suggesting that OsCIPK30 may be involved in biotic stress. This provides further evidence that OsCIPK genes are activated in response to both abiotic and biotic stresses.

CIPK proteins can activate kinase activity though interacting with calcineurin-B-like (CBL) proteins and form plant-specific Ca2+ sensor-effector modules (Yang and Poovaiah, 2003; Luan, 2009; Weinl and Kudla, 2009). There is some evidence showing that some components of this module are endogenous RNA silencing regulators that can be recruited by some plant viruses, such as the *Tobacco etch virus* (TEV), *Tomato golden mosaic virus* (TGMV), and *Tomato yellow leaf curl China virus* (TYLCCNV) (Anandalakshmi et al., 2000; Chung and Sunter, 2014; Li et al., 2014). Since NS3 has been reported as a viral suppressor of RNA silencing (VSR), we hypothesize that NS3 could recruit OsCIPK30 to perform its VSR function. However, this hypothesis remains highly speculative until the mechanism of the interaction between these two proteins is identified. We tried to use a co-localization assay to investigate the relationship between NS3 and OsCIPK30 (Supplementary Figure 1). The assays showed that NS3, OsCIPK30 localized in both the cytoplasm and nucleus when they were expressed in tobacco leaf epidermal cells alone. We also observed that the localization of OsCIPK30 was not obviously different from that when co-expressed with NS3, which indicated that the localization of OsCIPK30 and NS3 was not affected by each other.

Compared with WT, OsCIPK30-overexpressing rice induced high expression of pathogenesis-related genes in infected rice plants (**Figure 6**). The production and accumulation of pathogenesis-related proteins in plants in response to biotic stresses is well-known and is considered a crucial mechanism for plant defense (van Loon et al., 2006; Sels et al., 2008). For example, the accumulation of OsPR1a was enhanced with the treatment of protein phosphatase inhibitor cantharidin; the expression of PR-4b from *Theobroma cacao* is increased after infection in resistant genotypes, while in the susceptible genotypes the expression was only activated at the final stages of infection; OgPR1a from *Oryza grandiglumis* conferred disease resistance on *Arabidopsis* to bacterial and fungal infections (Agrawal et al., 2000; Pereira Menezes et al., 2014; Shin et al., 2014). Our results support and enrich the above opinions and indicate that the overexpression of OsCIPK30 induces the high expression of pathogenesis-related genes to enhance tolerance to RSV.

## Conclusion

Our results showed that the elevation of the pathogenesis-related genes occurs upon OsCIPK30 sensing RSV infection. The findings therefore provide new insight into the molecular mechanism underlying resistance to RSV disease.

## Author Contributions

YZ and TZ conceived and designed the experiments. ZL, XL, and FS performed the experiments. ZL and XL analyzed the data. ZL wrote and revised the paper.

## Conflict of Interest Statement

The authors declare that the research was conducted in the absence of any commercial or financial relationships that could be construed as a potential conflict of interest.

## Supplementary Material

DATA SHEET 1 Primers used in this study.
