# Supplementary material for: Overexpression of OsCIPK30 Enhances Plant Tolerance to Rice stripe virus
Source: Front Microbiol. 2017 Nov 24;8:2322. doi: 10.3389/fmicb.2017.02322 (PMC5705616; doi:10.3389/fmicb.2017.02322)
Supplement: Supplementary file 2 [file Presentation_1.PPTX]

## Slide 1
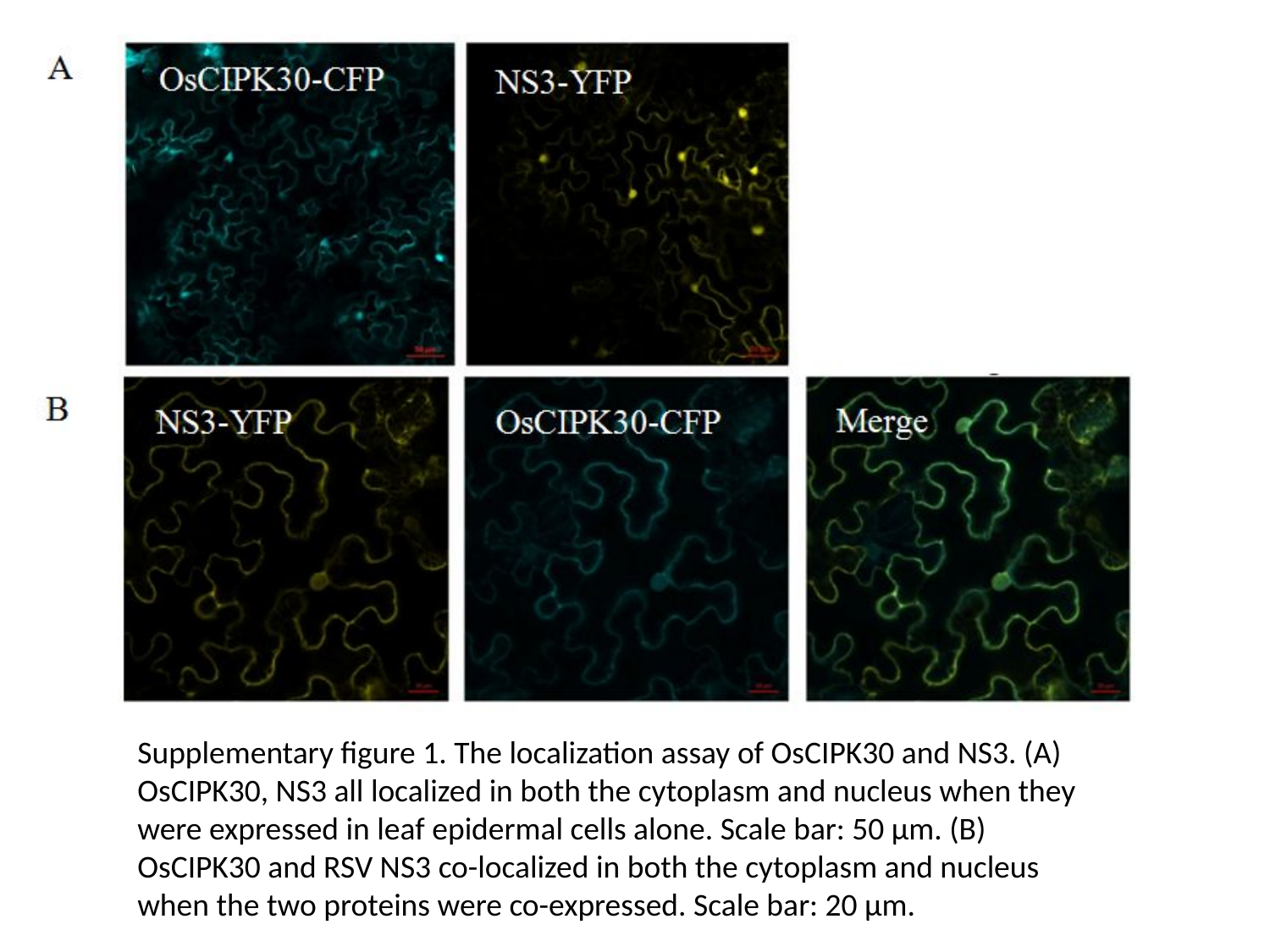

Supplementary figure 1. The localization assay of OsCIPK30 and NS3. (A) OsCIPK30, NS3 all localized in both the cytoplasm and nucleus when they were expressed in leaf epidermal cells alone. Scale bar: 50 μm. (B) OsCIPK30 and RSV NS3 co-localized in both the cytoplasm and nucleus when the two proteins were co-expressed. Scale bar: 20 μm.
